# Evaluation of Fifteen Algorithms for the Resolution of the Electrocardiography Imaging Inverse Problem Using *ex-vivo* and *in-silico* Data

**DOI:** 10.3389/fphys.2018.01708

**Published:** 2018-11-29

**Authors:** Amel Karoui, Laura Bear, Pauline Migerditichan, Nejib Zemzemi

**Affiliations:** ^1^Institute of Mathematics, University of Bordeaux, Bordeaux, France; ^2^INRIA Bordeaux Sud-Ouest, Bordeaux, France; ^3^IHU Lyric, Bordeaux, France

**Keywords:** inverse problem, Tikhonov regularization, L1-norm regularization, regularization parameter, method of fundamental solutions, finite element method, generalized singular value decomposition, pacing site localization

## Abstract

The electrocardiographic imaging inverse problem is ill-posed. Regularization has to be applied to stabilize the problem and solve for a realistic solution. Here, we assess different regularization methods for solving the inverse problem. In this study, we assess (i) zero order Tikhonov regularization (ZOT) in conjunction with the Method of Fundamental Solutions (MFS), (ii) ZOT regularization using the Finite Element Method (FEM), and (iii) the L1-Norm regularization of the current density on the heart surface combined with FEM. Moreover, we apply different approaches for computing the optimal regularization parameter, all based on the Generalized Singular Value Decomposition (GSVD). These methods include Generalized Cross Validation (GCV), Robust Generalized Cross Validation (RGCV), ADPC, U-Curve and Composite REsidual and Smoothing Operator (CRESO) methods. Both simulated and experimental data are used for this evaluation. Results show that the RGCV approach provides the best results to determine the optimal regularization parameter using both the FEM-ZOT and the FEM-L1-Norm. However for the MFS-ZOT, the GCV outperformed all the other regularization parameter choice methods in terms of relative error and correlation coefficient. Regarding the epicardial potential reconstruction, FEM-L1-Norm clearly outperforms the other methods using the simulated data but, using the experimental data, FEM based methods perform as well as MFS. Finally, the use of FEM-L1-Norm combined with RGCV provides robust results in the pacing site localization.

## 1. Introduction

The non-invasive electrocardiographic imaging (ECGI) is an imaging technique that allows one to non-invasively reconstruct the electrical activity of the heart using electrocardiograms and a patient specific heart-torso geometry. This clinical tool is used by electrophysiologists to understand the mechanisms underlying arrhythmias and to localize targets for ablation therapy, such as for atrial fibrillation (Haissaguerre et al., 2013; Rudy, 2013). This technology is based on a mathematical relationship defining the propagation of the electrical activity between the heart and the torso surface Γ_*ext*_. Given the extracellular electrical potential *u*_*H*_ on the epicardial heart boundary Γ_*H*_, the distribution of the electrical potential *u*_*T*_ in the torso domain Ω_*T*_ and specifically at electrodes distributed on the body surface Γ_*ext*_, could be obtained by solving the following Laplace equation:

(1) $$\begin{array}{l} \{\begin{array}{lll} \nabla \;\cdot \;(\sigma_{T}\nabla u_{\text{T}})=0, & \text{in} & \Omega_{T},\\ \sigma_{T}\nabla u_{\text{T}}\;\cdot \;n_{\text{T}}=0, & \text{on} & \Gamma_{ext},\\ u_{\text{T}}=u_{\text{H}}, & \text{on} & \Gamma_{H}. \end{array} \end{array}$$

where σ_*T*_ stands for the torso conductivity tensor and *n*_*T*_ is the outward unit normal to the torso external boundary Γ_*ext*_. This is what we call a forward problem. Now, given a body surface potential distribution and knowing that the flux of potential over the body surface is zero, could we obtain the right distribution of the electrical potential on the heart surface? This is what we call an inverse problem in electrocardiography. In almost all of the works reported in the literature, the mathematical approach used for solving the inverse problem is based on a transfer matrix which has been first formulated by Barr et al. (1977). The transfer matrix can be computed using different approaches such as the finite element method (FEM) (Wang et al., 2010; Zemzemi et al., 2015) or the boundary elements method like in Stenroos and Haueisen (2008); Stenroos (2009); Schuler et al. (2017); Ghosh and Rudy (2009); Chamorro-Servent et al. (2017); Barr et al. (1977), the method of fundamental solutions (MFS) (Wang and Rudy, 2006) or mixed methods like the factorization of boundary value method (Bouyssier et al., 2015) or finite element with mixed element types (Wang et al., 2010). In this study, we are only interested in FEM and MFS. Using any of these numerical approaches, the governing Equation (1) can be reduced to a matrix-vector system:

(2) $$\begin{array}{l} Ax=b, \end{array}$$

where **A** is the transfer matrix, its form depends on the numerical method used. The vector **x** is either the unknown epicardial potentials on the surface of the heart in the case of the FEM or a vector of weighting coefficients from which it's possible to reconstruct the epicardial potential in the case of MFS. Finally, **b** represents either the body surface potentials (BSPs) for the first case or a concatenation of the BSPs and a null vector representing the non flux boundary condition for the second case.Generally, the inverse problem of electrocardiography is known to be ill-posed in the sense of Hadamard Hadamard (1923) which means that a small perturbation of the Cauchy data may lead to a high variation in the inverse solution. This could be explained at the discrete level by the ill-conditioning of the transfer matrix **A** and the measurement noise that we have in the vector **b**. To overcome this, a regularization approach is often used to solve Equation (2). However, this has led to a large variety of different inverse algorithms being developed. To date, few studies have attempted to compare the different methods available. Cheng et al. (2003) looked at different regularization methods and methods to compute the regularization parameter. Since this work, many new methods have been developed.

A recent work by Barnes and Johnston (2016) compares several regularization techniques but without changing either the regularization operator or the numerical method defining the transfer matrix. Finally, both of these studies were based purely on simulated data, and their applicability to experimental or clinical work is unknown.

In this work we compare not only different methods for computing the transfer matrix, but also different regularization operators and different methods for optimizing the regularization parameter to assess how they perform on two sets of data: simulated and experimental.

## 2. Methods

To date, the regularization approach most commonly used to solve the electrocardiographic imaging inverse problem is the Tikhonov regularization defined by the following objective function:

(3) $$\begin{array}{l} \underset{x}{\min}\left\{∥Ax-b{∥}^{2}+\lambda^{2}∥Lx{∥}^{2}\right\}, \end{array}$$

where **L** is the regularization operator, λ is the regularization parameter and ∥.∥ is the L2-norm. Here, **L** can be the identity matrix (zero-order) or an approximation operator of a potential's derivative form (first or second order). Independent of the numerical method used to compute the transfer matrix, the best way to analyze the different methods to computing the optimal regularization parameter is to use the GSVD of the couple {**A**, *L*} for first or second order Tikhonov regularization and the singular value decomposition of **A** for zero-order.

### 2.1. Generalized singular value decomposition

In the case where ***L*** = ***I***, we use the Singular Value Decomposition of the ***m*** × ***n*** transfer matrix **A**, where ***m*** ≥ ***n***, ***m*** is the number of torso nodes and ***n*** is the number of heart nodes. Following Hansen (1998), we decompose ***A*** as follows

(4) $$\begin{array}{l} A=U\Sigma V^{T}=\overset{n}{\underset{i=1}{\sum }}u_{i}\sigma_{i}v_{i}^{T}, \end{array}$$

where **U** is a ***m*** × ***n*** orthonormal matrix containing the left singular vectors of **A**,**V** is a ***n*** × ***n*** orthonormal matrix containing the right singular vectors of **A** and Σ is a *n*×*n* diagonal matrix with the singular values of **A** on its diagonal. Note that ***u***_***i***_, ***v***_***i***_ and σ_***i***_ are, respectively, the columns of **U**, **V** and the singular values of **A** arranged in a decreasing order. In terms of the singular value decomposition, the solution of the regularized problem expressed by:

(5) $$\begin{array}{l} \underset{x}{\min}\left\{∥Ax-b{∥}^{2}+\lambda^{2}∥x{∥}^{2}\right\}, \end{array}$$

can be written as (Hansen, 1998):

(6) $$\begin{array}{l} x=A^{†}b={\left(A^{T}A+\lambda^{2}I\right)}^{-1}A^{T}b=\overset{n}{\underset{i=1}{\sum }}\frac{\sigma_{i}^{2}}{\sigma_{i}^{2}+\lambda^{2}}\frac{u_{i}^{T}b}{\sigma_{i}}v_{i}. \end{array}$$

It can be shown that the two terms of (5) can be written as (Johnston and Gulrajani, 1997):

(7) $$\begin{array}{l} \rho_{1}\left(\lambda \right)=∥Ax-b{∥}^{2}=\overset{n}{\underset{i=1}{\sum }}\frac{\lambda^{4}\mu_{i}^{2}}{{\left(\lambda^{2}+\sigma_{i}^{2}\right)}^{2}}+∥r_{⊥}{∥}^{2} \end{array}$$

and

(8) $$\begin{array}{l} \eta_{1}\left(\lambda \right)=∥x{∥}^{2}=\overset{n}{\underset{i=1}{\sum }}\frac{\sigma_{i}^{2}\mu_{i}^{2}}{{\left(\lambda^{2}+\sigma_{i}^{2}\right)}^{2}}, \end{array}$$

where $∥r_{⊥}{∥}^{2}=∥Ax_{LSS}-b{∥}^{2}$ is the residual of the least squares solution ***x***_***LSS***_ and $\mu_{i}=u_{i}^{T}b$.

In the case where ***L*** ≠ ***I***, the Generalized Singular Value Decomposition of the pair **{***A, L***}** is defined by (Hansen, 2010):

(9) $$\begin{array}{l} A=PCZ^{-1},\;L=QSZ^{-1}, \end{array}$$

where **P** and **Q** are, respectively, ***m*** × ***n*** and ***n*** × ***n*** orthogonal matrices. **C** and **S** are ***m*** × ***n*** and ***n*** × ***n*** diagonal matrices satisfying ***C***^***T***^***C*** + ***S***^***T***^***S*** = ***I*** where ***diag***(***C***) = {σ_1_ … σ_***n***_} and ***diag***(***S***) = {ν_1_ … ν_***n***_}. Diagonal elements of **C** and **S** satisfy 0 ≤ σ_1_ ≤ … ≤ σ_***n***_ ≤ 1 and 1 ≥ ν_1_ ≥ … ≥ ν_***n***_ ≥ 0. The matrix **Z** is non singular. We define ${\overset{-}{\lambda }}_{i}=\frac{\sigma_{i}}{\nu_{i}}$ as the generalized singular values of the pair **{***A, L***}**.

Using the generalized singular value decomposition, the solution of the problem expressed by Equation (3) can be written as (Chung et al., 2014):

(10) $$\begin{array}{l} x^{*}=A^{\#}b={\left(A^{T}A+\lambda^{2}L^{T}L\right)}^{-1}A^{T}b=\overset{n}{\underset{i=1}{\sum }}\phi_{i}\frac{p_{i}^{T}b}{\sigma_{i}}z_{i}, \end{array}$$

where Φ is a ***n*** × ***n*** diagonal matrix containing the **filter factors** defined by:

(11) $$\begin{array}{l} \phi_{i}=\frac{{\overset{-}{\lambda }}_{i}^{2}}{{\overset{-}{\lambda }}_{i}^{2}+\lambda^{2}},\;\text{for }i=1\ldots n. \end{array}$$

It can be shown that the two terms of (3) can be written in terms of generalized singular values as (Chung et al., 2014):

(12) $$\begin{array}{l} \rho_{2}\left(\lambda \right)=∥Ax^{*}-b{∥}^{2}=\overset{n}{\underset{i=1}{\sum }}{\left(\frac{\lambda^{2}}{{\overset{-}{\lambda }}_{i}^{2}+\lambda^{2}}\right)}^{2}{\left(p_{i}^{T}b\right)}^{2}+\overset{m}{\underset{i=n+1}{\sum }}{\left(p_{i}^{T}b\right)}^{2}, \end{array}$$

and (Ghista, 2012)

(13) $$\begin{array}{l} \eta_{2}\left(\lambda \right)=∥Lx^{*}{∥}^{2}=\overset{n}{\underset{i=1}{\sum }}{\left(\frac{{\overset{-}{\lambda }}_{i}}{{\overset{-}{\lambda }}_{i}^{2}+\lambda^{2}}\right)}^{2}{\left(p_{i}^{T}b\right)}^{2}. \end{array}$$

### 2.2. Regularization techniques

Several regularization techniques can be applied to the ill-posed inverse problem of electrocardiography. In this study, we focus on two methods.

#### 2.2.1. Zero order tikhonov regularization

Using the zero order Tikhonov regularization, the objective function can be expressed by (5). This type of regularization places a constraint on the magnitude of the reconstructed epicardial potentials which is known to provide a smooth solution but may lead to the loss of meaningful information.

#### 2.2.2. L1-norm regularization of the current density over the heart surface

Previous studies have shown that using the L1-Norm can provide a better reconstruction when applied in different fields (Wolters et al., 2004; Bai et al., 2007; Ding and Hei, 2008). In this paper, we choose to apply the regularization scheme used in Ghosh and Rudy (2009). Here, we penalized the L1-Norm of the normal derivative of the solution. The potential normal derivative represents the distribution of electrical flux over the epicardial surface.

This will yield less smoothed potentials than zero-order Tikhonov. The use of current density in the regularization of the inverse problem in electrocardiography was first introduced by Khoury (1994) and proved to provide significant improvement in the inverse problem.

The objective function using L1-Norm based regularization is given by:

(14) $$\begin{array}{l} \underset{x}{\min}∥Ax-b∥+\lambda^{2}∥\nabla x.n_{H}{∥}_{1}, \end{array}$$

where **n**_***H***_ is the outward unit normal to the epicardium surface.Using the Finite Element Method, and thanks to the linearity of the solution of problem (1) to its boundary conditions, we can define the Dirichlet-To-Neumann operator ***D*** satisfying:

(15) $$\begin{array}{l} \left(\begin{array}{c} \frac{\partial u_{T}}{\partial n}(p_{1})\\ \vdots \\ \frac{\partial u_{T}}{\partial n}(p_{n}) \end{array}\right)=D\left(\begin{array}{c} x_{1}\\ \vdots \\ x_{n} \end{array}\right), \end{array}$$

where ***D*** is an n-by-n matrix and the points (***p***_**1**_, ***p***_**2**_, …, ***p***_***n***_) are the coordinate tuples of the heart mesh vertices. Note that the operator D is different from the gradient over the surface used for the total variation regularization. In fact the gradient of x over the heart surface (∇_Γ_***H***__***x***) is the tangential component of electrical potential gradient (∇***u***_***T***_), whereas ***Dx*** is its normal component. Thus one could write the 3D gradient of the potential on the epicardial boundary as the sum of both components (∇*u*_***T***_ = (∇_Γ_***H***__***x*** + ***Dx***). The operator ∇_Γ_***H***__ depends only on the epicardial surface Γ_***H***_, whereas, ***D*** depends on the whole torso domain Ω. The objective function (14) can be expressed as follows:

(16) $$\begin{array}{l} \underset{x}{\min}∥Ax-b∥+\lambda^{2}∥Dx{∥}_{1}. \end{array}$$

The L1-Norm regularization of the current density leads to a non-linear problem. Following Karl (2005), we can smoothly approximate the L1-Norm of the derivative by:

(17) $$\begin{array}{l} ∥Dx{∥}_{1}=\overset{n}{\underset{i=1}{\sum }}|{\left\lfloor Dx\right\rfloor }_{i}|\approx \overset{n}{\underset{i=1}{\sum }}\sqrt{|{\left\lfloor Dx\right\rfloor }_{i}{|}^{2}+\beta }, \end{array}$$

with β a small constant satisfying β > **0** and ⌊***Dx***⌋_*i*_ the *i*^*th*^ component of the vector ***Dx***.

This approximation leads to an interesting formulation of the L1-Norm regularization problem in the form of a set of equations whose resolution as β → 0 gives an estimate of the solution of (16). The linear problem to be solved is then:

(18) $$\begin{array}{l} \left[A^{T}A+\lambda^{2}D^{T}W_{\beta }\left(x\right)D\right]x=A^{T}b, \end{array}$$

where ***W***_β_(***x***) is a diagonal matrix called **weight matrix**, expressed by:

(19) $$\begin{array}{l} W_{\beta }\left(x\right)=\frac{1}{2}diag\left[\frac{1}{\sqrt{|{\left\lfloor Dx\right\rfloor }_{i}{|}^{2}+\beta }}\right]. \end{array}$$

We notice that (19) has an effect on the variation of the normal derivative penalty. In fact, when the local normal derivative is too small, the weight goes to larger values imposing greater smoothness on the solution. When the local normal derivative is large, the weight goes to small values allowing larger gradients in the solution in these regions.

The above formulation can be further simplified in a way that it can be seen as a first-order Tikhonov regularization. In fact, thanks to the diagonality of ***W***_β_(***x***), (18) can be written such that:

(20) $$\begin{array}{l} \left[A^{T}A+\lambda^{2}D^{T}{\left(\sqrt{W_{\beta }\left(x\right)}\right)}^{T}\left(\sqrt{W_{\beta }\left(x\right)}\right)D\right]x=A^{T}b, \end{array}$$

which leads to:

(21) $$\begin{array}{l} \left[A^{T}A+\lambda^{2}{\tilde{D}}^{T}\left(x\right)\tilde{D}\left(x\right)\right]x=A^{T}b, \end{array}$$

where $\tilde{D}\left(x\right)=\sqrt{W_{\beta }\left(x\right)}D$.

Computationally, the Equation (21) is still non-linear since the weighting matrix ***W***_β_(*x*) depends on the solution ***x***. To overcome this constraint, we suggest to use the zero-order Tikhonov solution instead of the solution itself. Thus, the problem that we solve is

(22) $$\begin{array}{l} \left[A^{T}A+\lambda^{2}{\tilde{D}}^{T}\left(x_{0}\right)\tilde{D}\left(x_{0}\right)\right]x=A^{T}b, \end{array}$$

where ***x***_**0**_ is the zero-order Tikhonov solution determined by the Finite Element Method.

### 2.3. Methods for choosing regularization parameter

In this section, we detail the formulation of several methods used for choosing the optimal regularization parameter in terms of, both, the singular value decomposition in the case of the zero-order Tikhonov regularization and the generalized singular value decomposition in the case of L1-Norm regularization of the current density treated as a first-order Tikhonov regularization. It's fundamental for a good regularization parameter λ to satisfy the **Discrete Picard Condition** (DPC) (Hansen, 1990). In other words, this means that the singular values σ_***i***_ and the generalized singular values $\overset{-}{\lambda }$ that are greater than λ must decay to zero slower than the corresponding $\left|u_{i}^{T}b\right|$ and $\left|p_{i}^{T}b\right|$, respectively.

#### 2.3.1. U-curve

The U-Curve is a plot of the sum of the inverse of η_1_(λ) (respectively, η_2_(λ)) and the inverse of the corresponding residual ρ_1_(λ) (respectively, ρ_2_(λ)) in the case where ***L*** = ***I*** (respectively, ***L*** ≠ ***I***), in terms of λ on a log-log scale:

(23) $$\begin{array}{l} \{\begin{array}{cc} Ucurve(\lambda )=\frac{1}{\rho_{1}(\lambda )}+\frac{1}{\eta_{1}(\lambda )},\; & \text{if }L=I,\\ \; & \;\\ Ucurve(\lambda )=\frac{1}{\rho_{2}(\lambda )}+\frac{1}{\eta_{2}(\lambda )},\; & \text{if }L\neq I. \end{array} \end{array}$$

The U-Curve method was proposed by Krawczyk-Stańdo and Rudnicki (2007) and Krawczyk-Stańdo and Rudnicki (2008) and tested by Krawczyk-Stańdo and Rudnicki (2007), Krawczyk-Stańdo and Rudnicki (2008), and Yuan et al. (2010) for the selection of the regularization parameter in the inverse problem. These works presented the method as a tool to determine the interval to which the regularization parameter belongs, providing a better computing efficiency.

According to Krawczyk-Stańdo and Rudnicki (2007) results, ***Ucurve***(λ) is strictly decreasing on the interval $\left[0,{\delta_{n}}^{2/3}\right]$ and strictly increasing on the interval $\left[\delta_{1}^{2/3},\infty \right]$ where δ_1_ and δ_*n*_ are, respectively, the biggest and the smallest singular values (generalized singular value in the case where ***L*** ≠ ***I***). Thus, ***Ucurve***(λ) reaches a local minimum in the interval $\left[\delta_{n}^{2/3},\delta_{1}^{2/3}\right]$. If we have at least one non-zero singular value, we can ensure the uniqueness of the ***Ucurve***(λ) minimizer, λ_***u***_, the optimum value of λ.

#### 2.3.2. ADPC

As mentioned above, the optimal regularization parameter should satisfy the DPC. Therefore, ADPC is a regularization parameter choice method based on this condition. The idea is to look for the last index ***i*** before the DPC is no longer satisfied (Chamorro-Servent et al., 2017). This means before σ_***i***_ becomes smaller than $\left|u_{i}^{T}b_{t}\right|$ in a log-log scale where ***t*** is time. For the sake of simplification, $\log\left(\left|u_{i}^{T}b_{t}\right|\right)$ is fitted by a polynomial $p_{t}\left(i,\log\left(\left|u_{i}^{T}b_{t}\right|\right)\right)$ of degree 5 to 7. Then, for each ***p***_***t***_, we seek for α_***t***_ = σ_***maxi***_ such that log(σ_***i***_) ≥ ***p***_***t***_. The ADPC regularization parameter is then λ = ***median***(α_***t***_).

#### 2.3.3. CRESO

The Composite REsidual and Smoothing Operator (CRESO) method was introduced by Colli-Franzone et al. (1985). It chooses the parameter that corresponds to the first local maximum of the derivative of the difference between the constraint term and the residual term with respect to λ^2^.

(24) $$\begin{array}{l} \{\begin{array}{cc} C(\lambda )=\frac{d}{d(\lambda^{2})}(\lambda^{2}\eta_{1}(\lambda )-\rho_{1}(\lambda )),\; & \text{if }L=I,\\ \; & \;\\ C(\lambda )=\frac{d}{d(\lambda^{2})}(\lambda^{2}\eta_{2}(\lambda )-\rho_{2}(\lambda )),\; & \text{if }L\neq I. \end{array} \end{array}$$

In terms of the singular value decomposition, this can be written as (Johnston and Gulrajani, 1997; Ghista, 2012):

(25) $$\begin{array}{l} \{\begin{array}{cc} C(\lambda )=\sum_{i=1}^{n}\frac{\sigma_{i}^{2}\mu_{i}^{2}(\sigma_{i}^{2}-3\lambda^{2})}{{\left(\sigma_{i}^{2}+\lambda^{2}\right)}^{3}},\; & \text{if }L=I,\\ \; & \;\\ C(\lambda )=\sum_{i=1}^{n}\frac{{\bar{\lambda }}_{i}^{2}\alpha_{i}^{2}({\bar{\lambda }}_{i}^{2}-3\lambda^{2})}{{\left({\bar{\lambda }}_{i}^{2}+\lambda^{2}\right)}^{3}},\; & \text{if }L\neq I. \end{array} \end{array}$$

where $\alpha_{i}=p_{i}^{T}b,\;i=1\ldots n$.

#### 2.3.4. GCV

The Generalized-Cross Validation (GCV) (Wahba, 1977) is also a well-known method to choose the regularization parameter. It provides the optimal value of λ by minimizing the function:

(26) $$\begin{array}{l} \{\begin{array}{cc} G(\lambda )=\frac{\rho_{1}(\lambda )}{{\left[Trace(I-AA^{†})\right]}^{2}},\; & \text{if }L=I,\\ \; & \;\\ G(\lambda )=\frac{\rho_{2}(\lambda )}{{\left[Trace(I-AA^{\#})\right]}^{2}},\; & \text{if }L\neq I. \end{array} \end{array}$$

The function ***G***(λ) is, according to ***Wahba*** (Wahba, 1977), equal to the weighted linear combination of the ***m*** prediction errors by leaving out, in each time, the ***k***^***th***^ data point, ***k*** = 1..***m*** and resolving the inverse problem by the use of the ***m*** − 1 remaining data points. The idea is that the optimum of the regularization parameter provides the best prediction of a measurement as a function of the others. In terms of singular value decomposition, ***G***(λ) is expressed by (Wahba, 1977; Chung et al., 2014):

(27) $$\begin{array}{l} \{\begin{array}{l} \begin{array}{cc} G(\lambda )=\frac{\sum_{i=1}^{n}\frac{\lambda^{4}\mu_{i}^{2}}{{\left(\sigma_{i}^{2}+\lambda^{2}\right)}^{2}}+∥r_{⊥}{∥}^{2}}{{\left(m-\sum_{i=1}^{n}\frac{\sigma_{i}^{2}}{\sigma_{i}^{2}+\lambda^{2}}\right)}^{2}},\; & \text{if }L=I,\\ \; & \;\\ G(\lambda )=\frac{\sum_{i=1}^{n}\frac{\lambda^{4}\alpha_{i}^{2}}{{\left({\bar{\lambda }}_{i}^{2}+\lambda^{2}\right)}^{2}}+\sum_{i=n+1}^{m}\alpha_{i}^{2}}{{\left(m-\sum_{i=1}^{n}\frac{{\bar{\lambda }}_{i}^{2}}{{\bar{\lambda }}_{i}^{2}+\lambda^{2}}\right)}^{2}},\; & \text{if }L\neq I. \end{array} \end{array} \end{array}$$

It's known that the GCV method has good asymptotic properties as ***n*** → ∞ (Craven and Wahba, 1978; Golub et al., 1979; Lukas, 1993). However, it may not be reliable for small or medium values of *n* and can give values of λ that are too small resulting in a very noisy regularized solution.

#### 2.3.5. RGCV

In Lukas (2006), a new method called Robust GCV (RGCV) is proposed and proved to be more reliable than GCV for small values of ***n*** and generally more accurate. The RGCV estimate is defined by the minimizer of the following function:

(28) $$\begin{array}{l} R\left(\lambda \right)=\left[\gamma +\left(1-\gamma \right)\xi \left(\lambda \right)\right]G\left(\lambda \right), \end{array}$$

where ***G***(λ) is given by (26) and ξ(λ) is defined as:

(29) $$\begin{array}{l} \{\begin{array}{cc} \xi (\lambda )=Trace[{\left(AA^{†}\right)}^{2}]=\sum_{i=1}^{n}\frac{\sigma_{i}^{4}}{{\left(\lambda^{2}+\sigma_{i}^{2}\right)}^{2}},\; & \text{if }L=I,\\ \; & \;\\ \xi (\lambda )=Trace[{\left(AA^{\#}\right)}^{2}]=\sum_{i=1}^{n}\frac{{\bar{\lambda }}_{i}^{4}}{{\left(\lambda^{2}+{\bar{\lambda }}_{i}^{2}\right)}^{2}}, & \text{if }L\neq I.\\ \; & \; \end{array} \end{array}$$

Here, γ is called a robustness parameter, γ ∈ [0, 1].

The RGCV method is based on the average influence $\frac{1}{m}\overset{m}{\underset{i=1}{\sum }}∥Ax_{\lambda }-Ax_{\lambda }^{\left[i\right]}{∥}^{2}$, where $∥Ax_{\lambda }-Ax_{\lambda }^{\left[i\right]}{∥}^{2}$ is a measure of the influence of the ***i***^***th***^ data point on the regularized solution. It's trivial that, when γ = 1, ***R***(λ) is reduced to ***G***(λ). It can be shown that the term (1 − γ)ξ(λ) penalizes the too small values of λ. In fact, when λ → ∞, ξ(λ) → 0, so $\frac{1}{\gamma }R\left(\lambda \right)$ becomes equivalent to *G*(λ). Otherwise, if λ → 0, ξ(0) = ***n***, so $\frac{1}{\gamma }R\left(\lambda \right)\gg G\left(\lambda \right)$ for small values of γ which means that the smaller γ, the more robust is the RGCV method (Lukas, 2006).

## 3. Experimental methods and simulation protocols

### 3.1. Data sets

**Figure 1:**
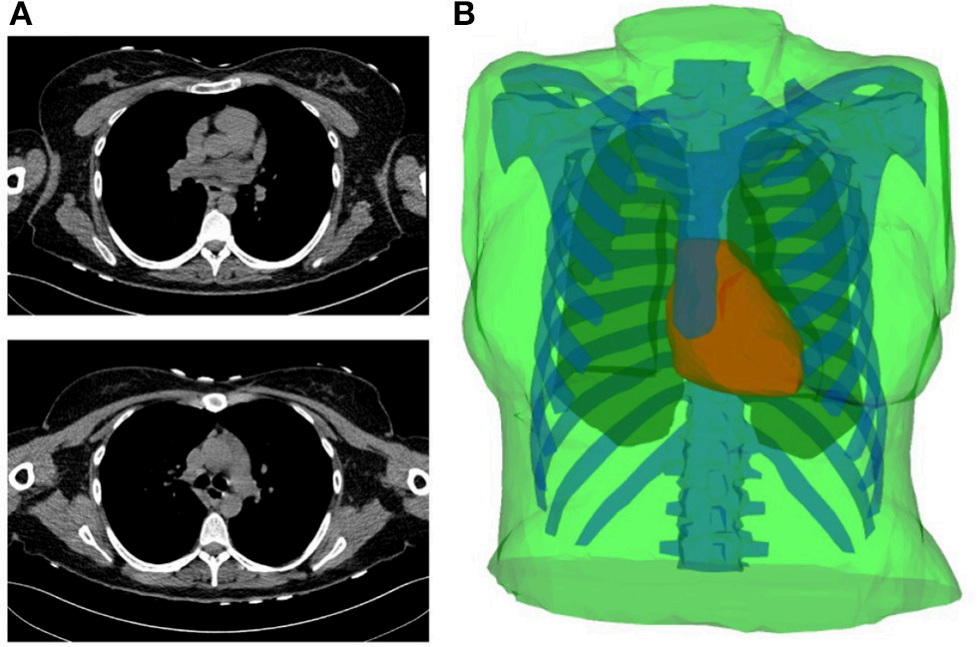
**(A)** Two slices of the CT-scan images. **(B)** Torso geometry showing the epicardium (heart-torso interface Σ) (red), lungs (yellow), bones (blue) and torso external boundary Γ_*ext*_ (green).

**Figure 2:**
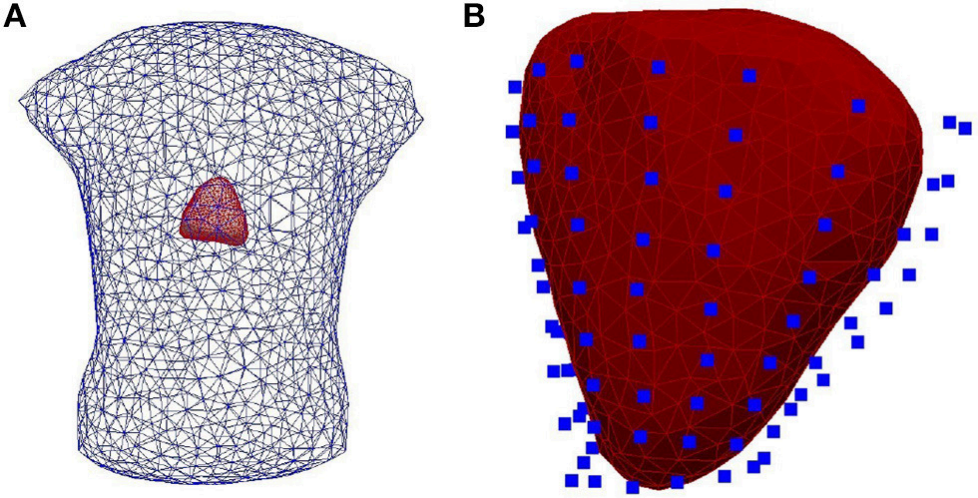
**(A)** The heart-human-shaped torso tank model used for the experimental data simulations. The heart consists of 761 nodes and 1,518 elements and the tank contains 1,177 nodes and 2,350 elements. **(B)** The heart geometry covered by the sock consisting of 108 electrodes (blue points).

ECGI reconstructions were performed on two different sets of data:

- Simulated data obtained by considering a realistic 3D heart-torso geometry segmented from CT-Scan images as illustrated in Figure 1 (see Zemzemi et al., 2014 for more details). The propagation of the electrical wave was computed using the monodomain reaction-diffusion model. The transmembrane currents used to compute the extracellular potential distribution throughout the torso were computed by solving a static bidomain problem in an homogeneous, isotropic torso model (Boulakia et al., 2010). Synchronized electrical potential on the epicardium and on the body surface were extracted in order to test the inverse methods. The torso mesh contained 2,873 nodes and the heart mesh 519 nodes.
- Experimental data were obtained using an *ex-vivo* pig heart perfused in Langendorff mode suspended into a human-shaped torso tank. The heart was paced by 2 ms pulses at 2 Hz, with constant current amplitudes 2x the diastolic threshold, on the left and right ventricular epicardial surface, mimicking ectopic activity. Epicardial ventricular electrograms were recorded using a 108-electrode sock (of which 93 were used) simultaneously with torso potentials from 128 electrodes embedded in the tank surface as it appears in Figure 2.
  Tank and sock unipolar electrograms were recorded at 2 kHz (BioSemi, the Netherlands) and referenced to a Wilson's central terminal defined using tank electrodes. A multi-lead signal averaging algorithm was used to remove noise and non-synchronized p-waves on recordings. In most cases, retrograde VA conduction was present with P-waves only present during the non-analyzed ST-segment. The tank mesh contains 1,177 nodes and the epicardium 761 nodes. For the application of described inverse methods, potential recordings need to be available for all the mesh nodes. To do so, a linear interpolation was applied to the *ex-vivo* recordings. More details about the *ex-vivo* experimental protocol can be found in Bear et al. (2018).

For all the carried out tests using the L1-Norm regularization, β is kept fixed and equal to **10**^−5^.

### 3.2. Choice of the robustness parameter

**Figure 3:**
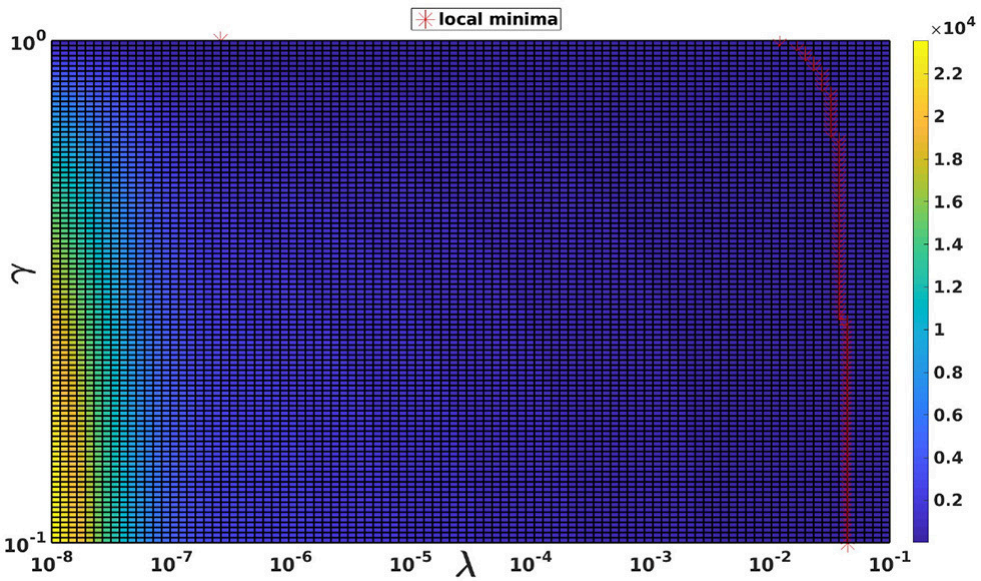
The RGCV criterion plotted in terms of λ and γ. The red markers are the grid points where RGCV(λ,γ) is minimum when γ is fixed.

The choice of γ for the RGCV tests is based on the study made by Barnes and Johnston (2016). In fact, they proved that applying RGCV with γ = **0** gives a good approximation of the optimal regularization parameter, especially when using realistic geometries and potential measures. To justify this choice, Figure 3 represents a plot of the RGCV criterion in terms of the parameters λ and γ where the color map defines the value of the RGCV function and the red marks correspond to the local minima. We observe that the local minima are almost reached at the same λ value except the case where γ = **1** corresponding to the GCV. For organization reasons, we present here only a graph realized using experimental data at a specific time step, but we observe the same behavior for all the other cases. This confirms the fact that for the inverse problem of electrocardiography, RGCV is not sensitive to γ when γ ∈ [**0, 0.5**].

### 3.3. Evaluation criteria

To assess the accuracy of the results obtained by the different approaches, we define the relative error (**RE**) and the correlation coefficient (**CC**):

(30) $$\begin{array}{l} RE=\sqrt{\frac{\sum_{i=1}^{n}{\left(x_{i}^{c}-x_{i}^{e}\right)}^{2}}{\sum_{i=1}^{n}{\left(x_{i}^{e}\right)}^{2}}} \end{array}$$

(31) $$\begin{array}{l} CC=\frac{\sum_{i=1}^{n}\left[x_{i}^{c}-\overset{-}{x^{c}}\right]\left[x_{i}^{e}-\overset{-}{x^{e}}\right]}{\sqrt{\sum_{i=1}^{n}{\left(x_{i}^{c}-\overset{-}{x^{c}}\right)}^{2}\sum_{i=1}^{n}{\left(x_{i}^{e}-\overset{-}{x^{e}}\right)}^{2}}} \end{array}$$

where *x*^*c*^ and *x*^*e*^ denote, respectively, the computed epicardial potential and the known one. *n* is either the number of epicardial nodes or the total number of time steps. In the first case, $\overset{-}{x^{c}}$ and $\overset{-}{x^{e}}$ are the spatial mean values of ***x***^***c***^ and ***x***^***e***^ over the ***n*** epicardial nodes. Otherwise, $\overset{-}{x^{c}}$ and $\overset{-}{x^{e}}$ are the temporal mean values of ***x***^***c***^ and ***x***^***e***^ over the ***n*** time steps. The means and the standard deviations of RE and CC are then computed and represented as bar graphs. The accuracy of pacing sites localization is measured by the geodesic distance between real and estimated pacing sites.

## 4. Results

### 4.1. Epicardial potential reconstruction

#### 4.1.1. Simulated data

First, we assessed regularization techniques and numerical methods using simulated data. The five regularization parameter choice criteria described above were assessed using all the suggested numerical methods: MFS, FEM-ZOT, and FEM-L1 which make 15 different algorithms.

**Figure 4:**
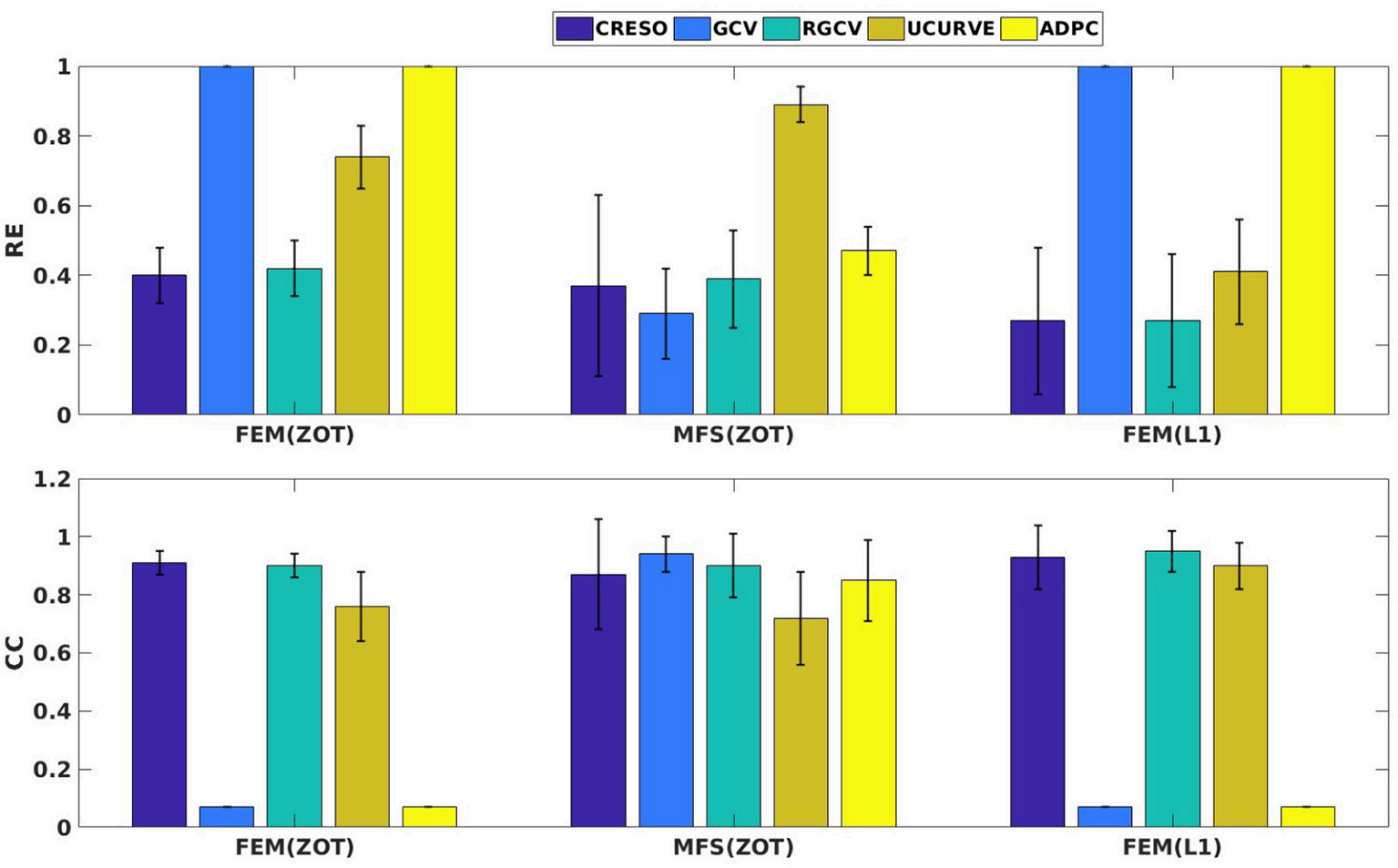
Bar graphs of means of relative errors and correlation coefficients with the standard deviations for simulated data.

Figure 4 presents the mean and the standard deviation of the spatial REs and CCs of the reconstructed potentials by the different numerical tests. For MFS, GCV gives the best estimation of the optimal regularization parameter in terms of relative error (**0.24** ± **0.15**) and correlation coefficient (**0.98** ± **0.04**). we notice an improvement by **10**% comparing to RGCV and CRESO methods. These 3 techniques outperform with different grades ADPC and U-Curve which seem to be unsuitable for MFS resolution.

For all the runned simulations using FEM, GCV and ADPC fail to compute the optimal regularization parameter. In fact, GCV tends to be flat for small values of λ which make it difficult to pick a minimum. RGCV is suggested to help with this difficulty. We observe here that it outperforms U-Curve by nearly 30% using the zero order Tikhonov and **20**% using the L1-norm regularization of the current density while it gives similar results to CRESO in terms of both spatial RE and CC.

**Figure 5:**
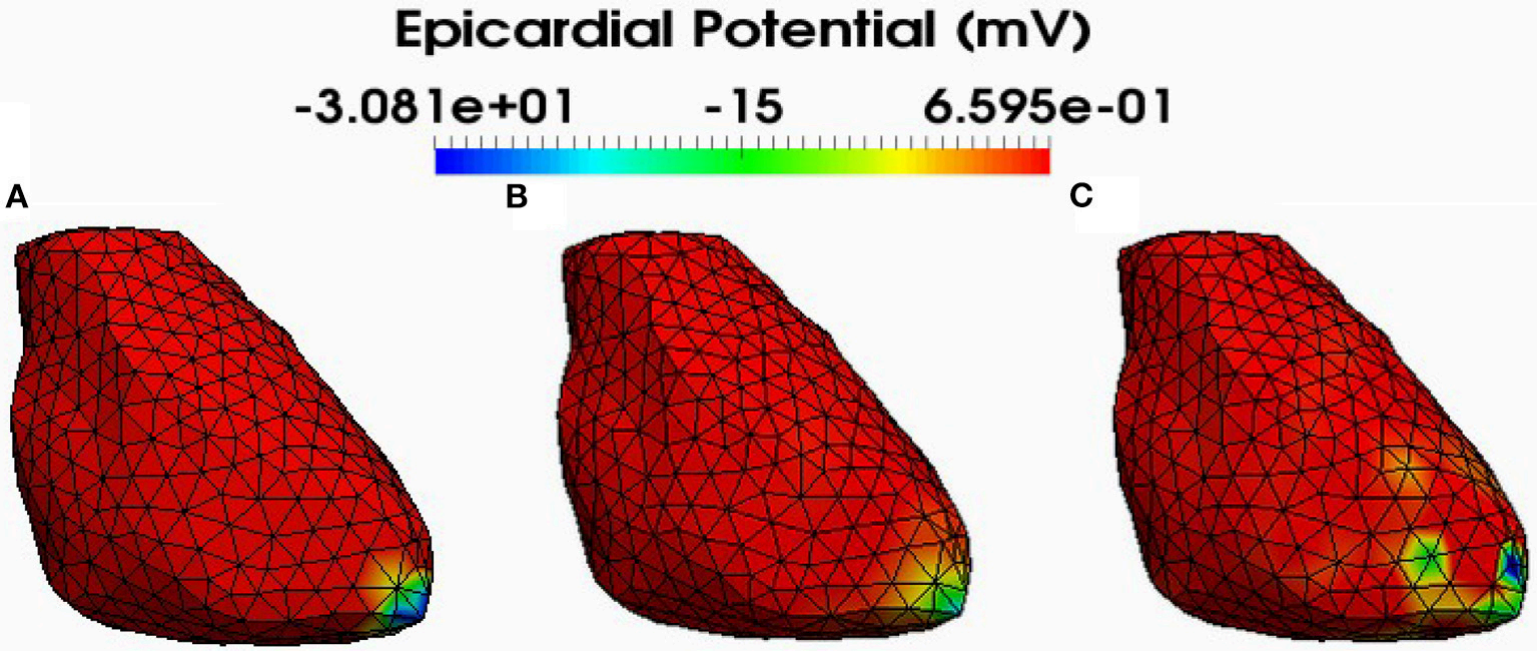
Simulated **(A)** and reconstructed epicardial potential distributions on the epicardium at the stimulation sample time using FEM-ZOT **(B)** with the optimal regularization parameter (RGCV), L1-Norm **(C)** with the optimal regularization parameter (RGCV).

**Figure 6:**
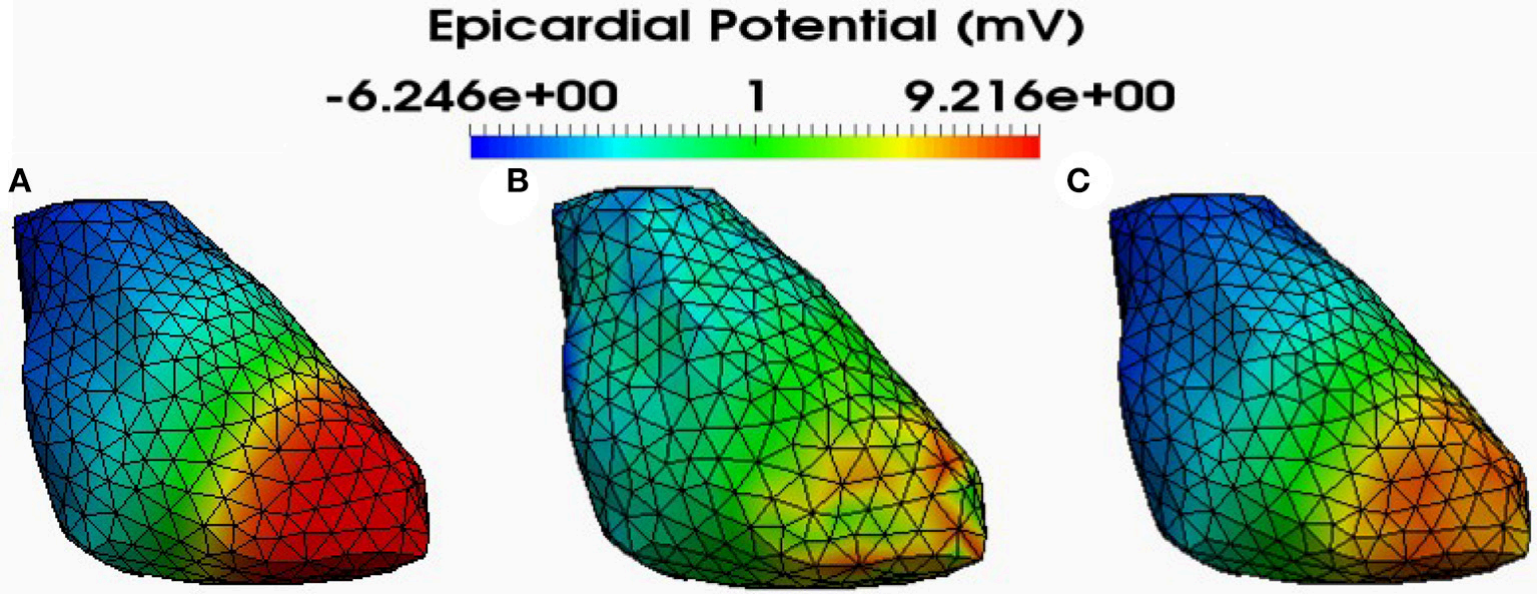
Simulated **(A)** and reconstructed epicardial potential distributions on the epicardium at 212ms after stimulation using FEM-ZOT **(B)** with the optimal regularization parameter (RGCV) and L1-Norm **(C)** with the optimal regularization parameter (RGCV).

Figure 4 shows also the accuracy of L1-norm regularization in the reconstruction of epicardial potential maps. We observe that it provides the minimum of mean relative error (**0.21 ± 0.2**) and the maximum of spatial correlation coefficient (**0.99 ± 0.04**). Figures 5, 6 show simulated epicardial potential maps (A) and reconstructed ones using FEM-ZOT (B) and FEM-L1-Norm (C) at the stimulation sample time and at 212 ms, after the electrical pacing leading to a reentry arrythmia, respectively. It can be seen that L1-Norm regularization provides a better reconstruction compared to the zero-order Tikhonov regularization especially on the regions where we have a potential leap. This fits exactly with the role of the L1-Norm regularization which is a better way to detect the gradient changes compared to Zero order Tikhonov.

#### 4.1.2. Experimental data

**Figure 7:**
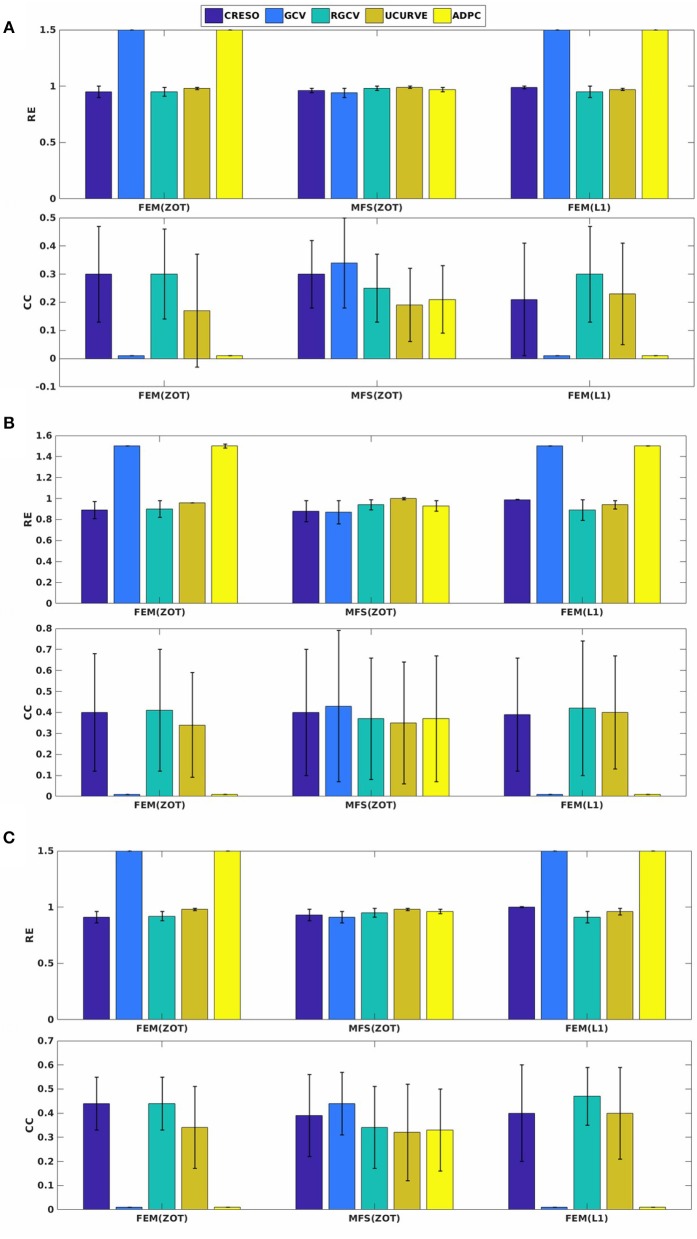
Spatial mean relative errors and correlation coefficients and their standard deviations for reconstructed epicardial potentials with all the algorithms for three paced rhythms: **(A)** Biv, **(B)** RV, and **(C)** LV.

Preprocessing of the experimental data revealed the existence of a few localized sites of ischemia produced due to electrode pressure on the epicardium. This produced monophasic action potential-like signals. These electrodes were identified when the potential was greater than a fixed threshold equal to **50**% of the maximum signal magnitude in the plateau phase, 250 ms after pacing. This choice is based on observations of the QT interval in order to eliminate the ischemic signals. This leads us to run two sets of comparisons, with all the working electrodes and after removing the above threshold electrodes. We observe that results after thresholding are better than those obtained with ischemic signals. For the sake of clarity, we present here only results after thresholding. Figure 7 shows the mean and standard deviation of spatial RE and CC. We observe a degradation of the metrics for the three models of experimental data (RV, LV, and BiV). This can be explained by different factors, the subject of section **4.4**. In Figure 7, we observe that using MFS, all the methods demonstrated similar trends in RE mean values. It shows also that GCV outperforms the other methods in terms of spatial correlation coefficient. For FEM, GCV and ADPC have always difficulties in computing the optimal value of the regularization parameter while RGCV, CRESO and U-Curve perform the same with a mean relative error near to **0.95** for all the three paced rhythms. Regarding the performance, there is not a clear difference among all the methods.

For the sake of completeness, statistical detailed results of RE and CC in time and space on the reconstructed potential for all cases are reported in the Supplementary Material.

### 4.2. Localization of pacing sites

**Figure 8:**
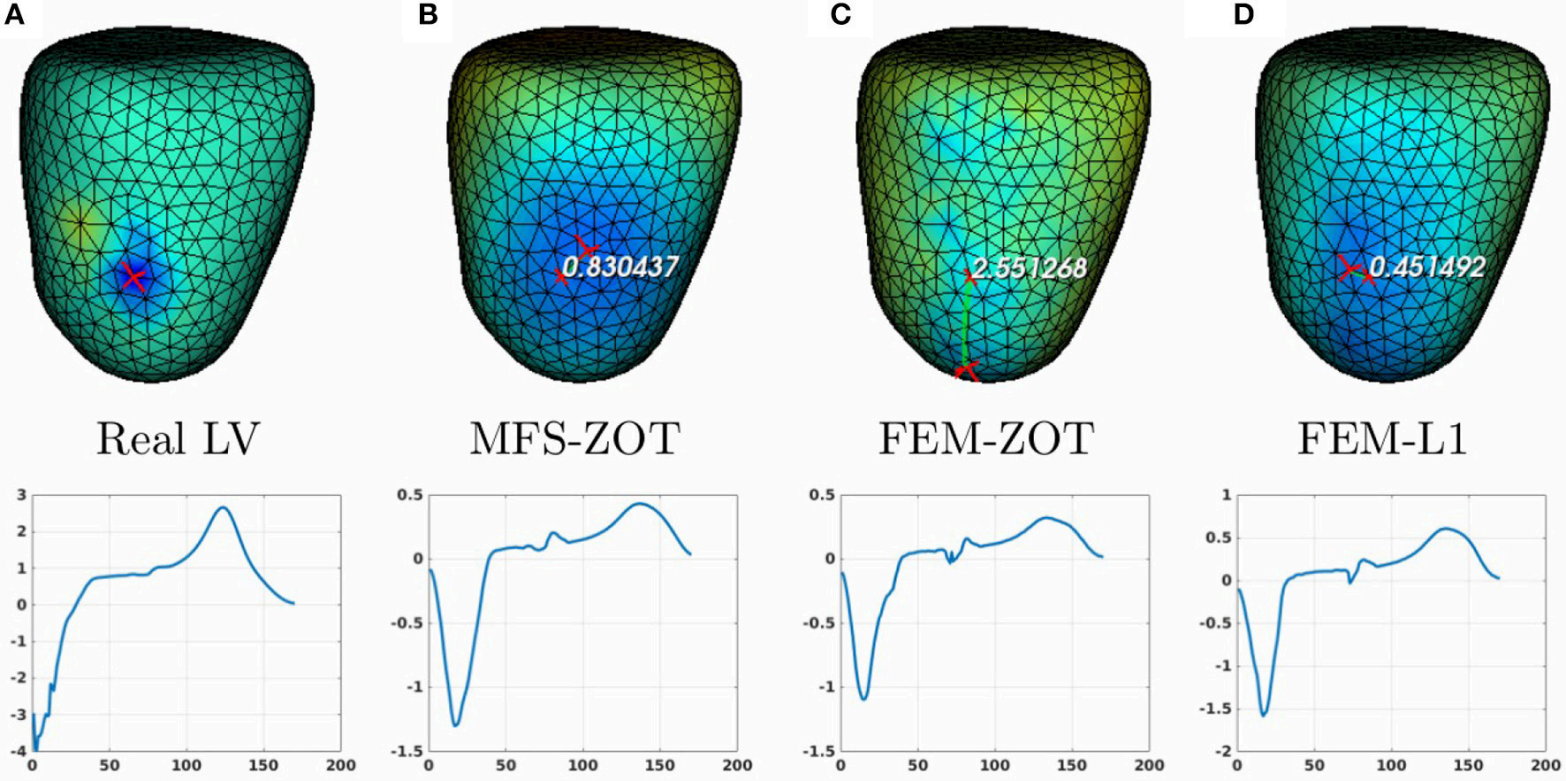
Real **(A)** and the estimated LV pacing sites (top) and its electrograms (bottom) using MFS-ZOT (B), FEM-ZOT **(C)**, and FEM-L1 **(D)**, respectively.

**Figure 9:**
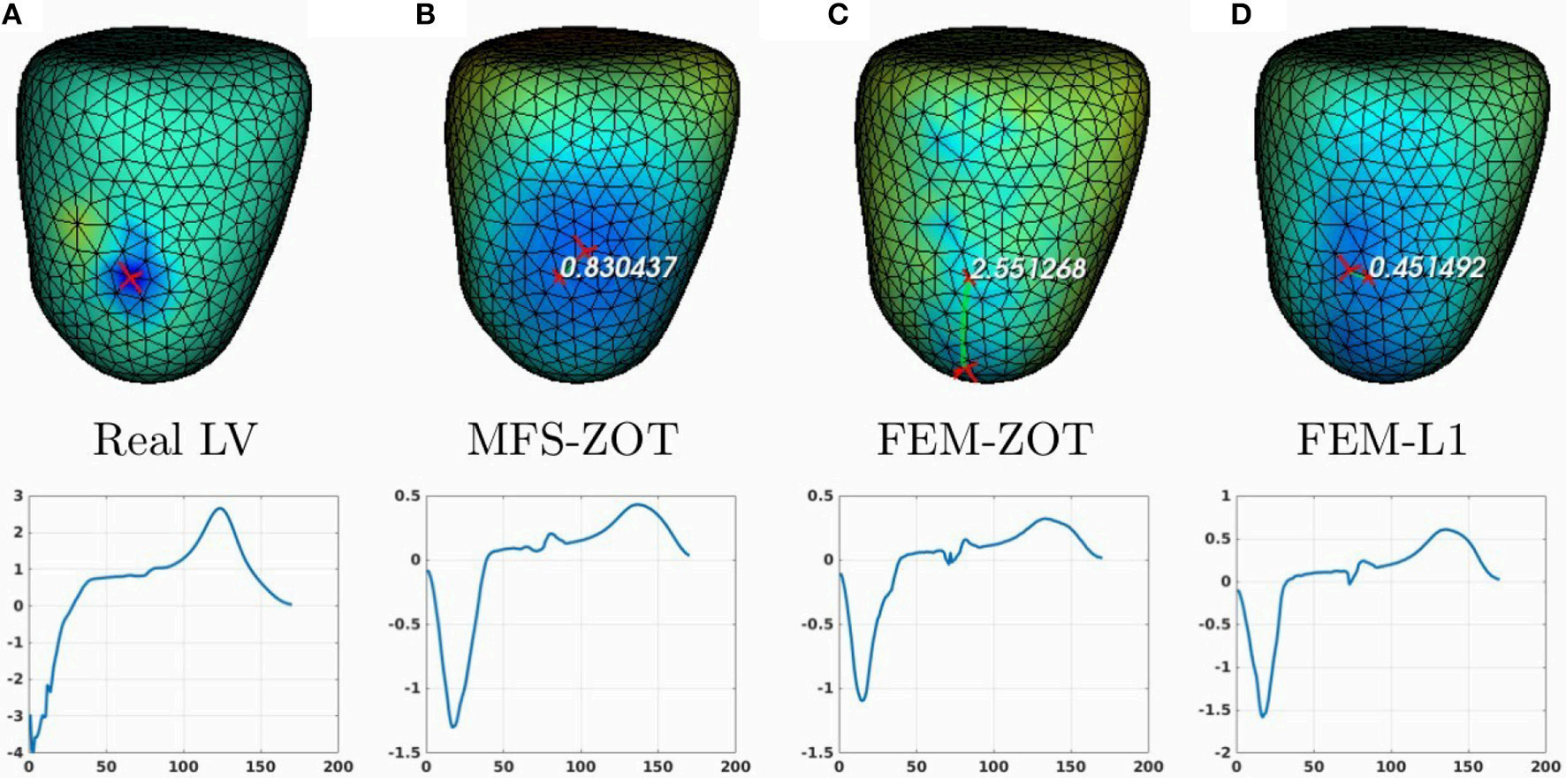
Real **(A)** and the estimated RV pacing sites (top) and its electrograms (bottom) using MFS-ZOT **(B)**, FEM-ZOT **(C)**, and FEM-L1 **(D)**, respectively.

For the localization of pacing sites, we used three different experiments, two of them provide LV, RV, and BiV pacing data sets and the other one has only RV and LV models. In summary, we have 3 cases of LV pacing, 3 cases of RV pacing and 2 cases of BiV pacing. In Figure 8 (respectively, Figure 9) (top), we show measured and reconstructed potential maps right at the pacing sample time in an LV-pacing (respectively, RV-pacing) case. The detected pacing sites are marked by bigger red crosses than the actual pacing site and the length of the green segment between them represents the geodesic distance. For the sake of comparison, only the simulation using the regularization parameter technique providing the better localization is selected for the figures. The case where the reconstructed epicardial potential do not allow us to extract the pacing sites are reported in Table 1 as non applicable (N.A) cases.

**Table 1:** Mean errors and standard deviations of localization of pacing sites for the 2 paced rhythms RV, LV using the 3 numerical methods MFS-ZOT, FEM-ZOT, and FEM-L1 combined with the regularization parameter choice methods.

		**CRESO**	**GCV**	**RGCV**	**UCurve**	**ADPC**
RV	MFS-ZOT	2.8 ± 1.2	2.4 ± 1.1	1.9 ± 0.9	2.4 ± 0.8	2.5 ± 0.8
	FEM-ZOT	2.7 ± 0.8	N.A	2.7 ± 0.9	2.0 ± 0.1	N.A
	FEM-L1	1.9 ± 0.5	N.A	1.8 ± 0.3	1.8 ± 0.4	N. A
LV	MFS-ZOT	1.7 ± 0.7	2.1 ± 0.3	2.0 ± 1.1	1.3 ± 0.6	2.1 ± 0.2
	FEM-ZOT	2.1 ± 0.4	N.A	2.8 ± 1.0	3.0 ± 0.2	N.A
	FEM-L1	1.3 ± 0.5	N.A	1.2 ± 0.6	1.3 ± 0.6	N.A
BiV	MFS-ZOT	2.5/*N*.*A*	2.3/1.5	0/*N*.*A*	2.3/*N*.*A*	2.7/2.0
	FEM-ZOT	1.8/*N*.*A*	N.A	1.8/2.1	2.5/N.A	N.A
	FEM-L1	2.5/N.A	N.A	1.3/1.4	1.4/N.A	N.A

*For BiV, values are the geodesic distances (LV/RV). N.A means that one could not extract the pacing site from the reconstructed signals. Highlighted values are the best localization errors*.

For the LV-pacing (respectively, RV-pacing) case, we observe that L1-norm regularization of the current density combined with RGCV provides the best localization with an error of **0.45*****cm*** (respectively, **2.15*****cm***). It outperforms FEM-ZOT **2.55*****cm*** (respectively, **2.16*****cm***) and MFS **0.83*****cm*** (respectively, **3.15*****cm***) that give similar approximations. We also plot in the bottom of the figure the time course of the electrical potential at the actual pacing site position detected from the measured data. For LV-pacing case, MFS, (respectively FEM-ZOT and FEM-L1) present temporal relative error and correlation coefficient equal to (**0.83, 0.72**) (respectively (**0.86, 0.75**), (**0.8, 0.72**)). For the RV-pacing case, MFS, (respectively FEM-ZOT and FEM-L1) present temporal relative error and correlation coefficient equal to (**1.05, 0.3**) (respectively (**1.12, 0.40**), (**1.01, 0.33**)).

For both LV and RV-pacing we observe that none of the methods is clear-cut.

**Figure 10:**
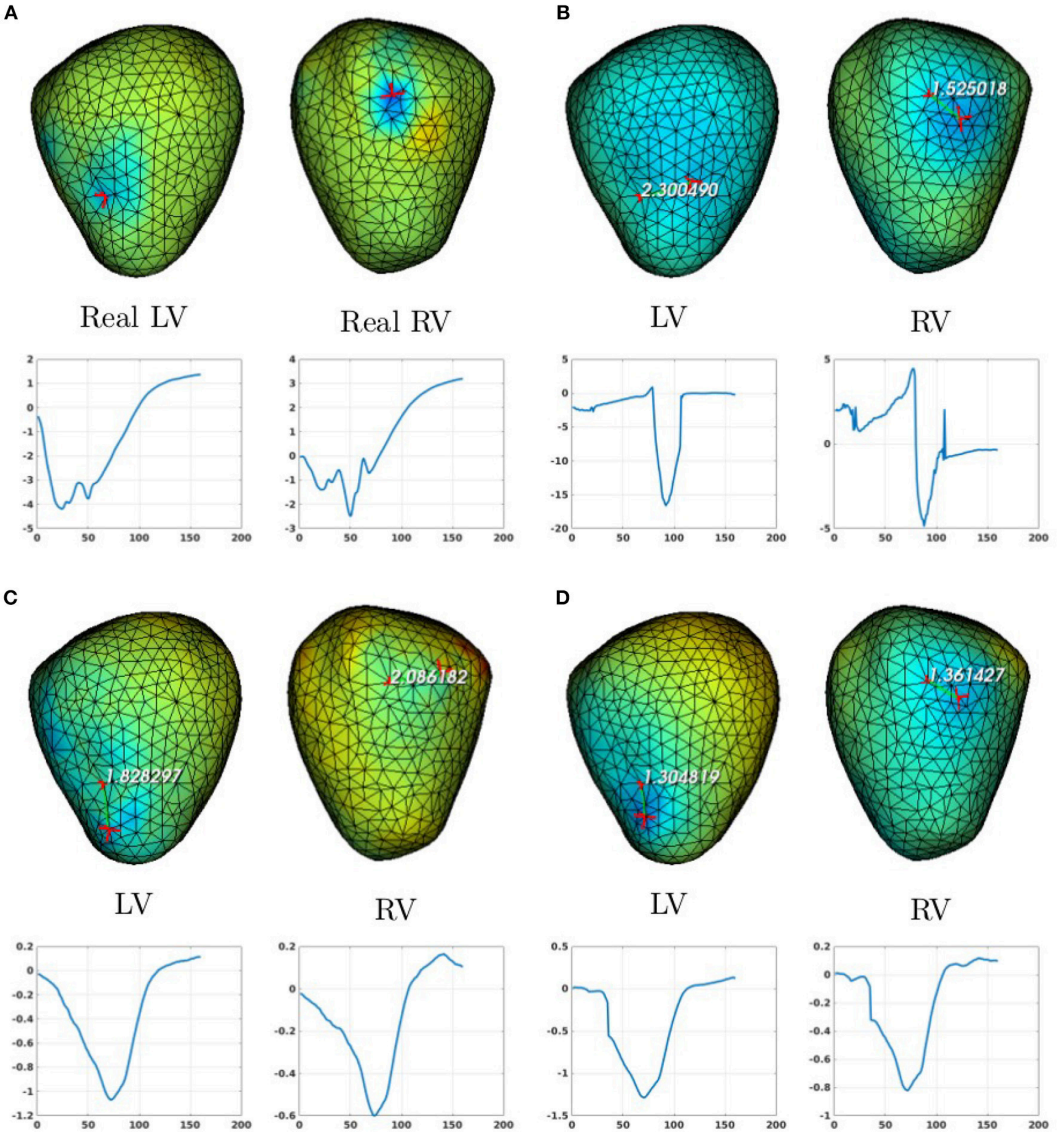
Real **(A)** and the estimated BiV pacing sites with its electrograms using the numerical methods **(B)** MFS-ZOT, **(C)** FEM-ZOT, and **(D)** FEM-L1. In each panel, LV and RV pacing sites (top) with their electrograms (bottom) are represented using the mentioned numerical method.

In the case of a bi-ventricular pacing (BiV), not all the methods were able to locate both pacing sites. Only MFS-ZOT combined with GCV, FEM-ZOT and FEM-L1 with RGCV succeed to detect the two pacing sites with more-less good accuracy. Figure 10 presents the real and estimated pacing sites and their electrograms for a BiV pacing rhythm for which all the methods work. The Figures 10B–D show the results for the BiV pacing sites. Errors of localization of the LV pacing site are **1.3*****cm*** for FEM-L1, **1.8*****cm*** for FEM-ZOT and **2.3*****cm*** for MFS. The bottom row of each panel represents the reconstructed electrograms in the real pacing sites using the specified method. The temporal relative errors and correlation coefficients for LV are (**0.80, 0.71**) using FEM-L1, (**0.86, 0.75**) with FEM-ZOT and (**0.83, 0.72**) using MFS. As shown in Figure 10B, MFS nearly fails to detect the left ventricular pacing site. The epicardial potential in the whole left ventricle is almost in the same range. For the RV pacing site, results are nearly the same as for the LV pacing site. The performance in terms of pacing site localization of the 15 algorithms on the set of the experimental data are reported in Table 1 where we provide the mean values and standard deviations of pacing sites localization errors for the three cases, LV, RV, and BiV. We remark that, L1-norm regularization of the current density combined with RGCV parameter choice method outperforms all the other methods with minimum errors and more stable standard deviations.

### 4.3. Limitations

#### 4.3.1. The imperfect knowledge of the transfer matrix

It's important to mention that in this work, the use of simulated data provides an optimal knowledge of the transfer matrix *A*, which is not the case of experimental data. It explains somehow the degradation of the results using the experimental data. To assess the impact of the transfer matrix, we computed a relative error defined by:

(32) $$\begin{array}{l} RE_{d}=\frac{∥Ax_{ex}-b∥}{∥b∥} \end{array}$$

where ***x***_***ex***_ is the exact solution whether it's the simulated epicardial potential or the measured one.

The ***RE***_***d***_ is almost equal to zero using the simulated transfer matrix. However, it increases for the experimental data to reach, for some time steps, ***RE***_***d***_ ≈ **0.9**. Although this issue is out of the scope of this paper, the degradation can be due to different factors like the measurement errors and geometrie's inaccuracy due to the fact that the heart is moving during the experiment, but also to the mathematical modeling of the physical phenomenon which is reduced to the Laplace equation. These hypotheses make the issue subject to further analyzes.

#### 4.3.2. Experimental protocols

Obviously, the experimental conditions have a very important impact on the quality of the data that we obtain from experiments. One of the limitations of this study is the dataset of epicardial signals. In fact, the experimental protocol described in Bear et al. (2018) indicates that the epicardial surface is not totally covered with electrodes which provides less information and biased results. Further studies should be done in this context. The protocols we have set until now do not include endocardial stimulation, this is one of the limitation of our work. Of course, if we have to evaluate the methods against endocardial and septal stimulations we have to make use of a W-shape geometry of the ventricles including endocardial, epicardial and septal surfaces instead of a nut-shape geometry that only represents the epicardial surface.

## 5. Discussion and conclusion

In this paper, we numerically assessed 15 different algorithms for the resolution of the inverse problem of electrocardiography based on the Generalized Singular Value Decomposition of the pair {Transfer matrix, Regularization matrix} combined with different regularization parameter choice methods. Although the L1-Norm of the normal derivative regularization method has been presented before (Khoury, 1994; Ghosh and Rudy, 2009) to solve the ECGI inverse problem, there are two novelties in this paper: First, the non quadratic scheme was solved using the generalized singular values decomposition, whereas, in Ghosh and Rudy (2009) authors use an iterative method. Second, the regularization method was combined with five regularization parameter choice methods to assess its performance on simulated and experimental data. In Barnes and Johnston (2016), authors used only ZOT regularization and compared results only on simulated data. In this paper and in the majority of the studies looking for the ECGI inverse solution, the problem is formulated in terms of electrical potential. There are other approaches, where the problem is formulated in terms of propagating wave front (Cuppen and Van Oosterom, 1984; Huiskamp and Greensite, 1997). In Van Dam et al. (2009), the activation and recovery times and the transmembrane potentials are constructed. Other approaches are interested in constructing directly dominant frequencies on the heart surface and torso surfaces (Pedrón-Torrecilla et al., 2016; Beltrán-Molina et al., 2017).

The evaluation of the different approaches studied in this paper is based on the reconstruction of the epicardial potential maps and the localization of pacing sites. For that, we used 3 different cardiac paced rhythms: left-ventricular, right-ventricular and bi-ventricular pacing.

Unlike the work presented by Barnes and Johnston (2016), this study considered two types of transfer matrices: MFS and FEM and two different approaches of regularization: zero-order Tikhonov and L1-Norm. This study demonstrated that, when using the MFS discretization approach, the GCV method is more appropriate and optimal than RGCV and the other parameter choice methods. Otherwise, for the FEM approach, the RGCV gives the best results using simulated data. But also, GCV and ADPC provide very weak results with FEM, this is mainly due to the fact that the minimization criteria in both cases chooses the regularization parameter λ at the lower bound of the provided interval.

However, for the experimental data, all the methods perform nearly the same with a slight difference in terms of both spatial and temporal relative error and correlation coefficient when comparing the epicardial potential distribution. We think that this is mainly due to the magnitude of the recorded potentials but also to the noise and other experimental uncertainties. Results show, also, that L1-Norm regularization of the potential normal derivative yields generally the best solution. For the purpose of benchmarking, the represented algorithms were evaluated against the data set used in the paper (Figuera et al., 2016). Results are reported in the Supplementary Material. They show similar performance for the sinus rhythm model using the L1-norm regularization of the current density. This last regularization has a better performance for the atrial fibrillation models compared to all the ZOT based methods but weaker results than the Bayesian approach (Serinagaoglu et al., 2005; Figuera et al., 2016). This should be subject of several further studies.

Regarding the pacing site localization, Table 1 show clearly that the estimation of pacing sites is more accurate using L1-norm regularization than other methods with minimum errors and less variance despite the fact that it depends of the epicardial potential reconstruction. This is due to the use of L1-Norm regularization that preserves the spatial gradient changes in the solution which is not the case for the L2-Norm regularization that tends to give smoother solutions. Despite the good performance of the methods in the case of LV and RV, they have faced difficulties in localizing two pacing sites for the BiV pacing and localize in some cases only one pacing site nearly equidistant to the two real ones. Some limitations of this study have been explored such as the imperfect knowledge of the transfer matrix and the noise in the ground truth data that could lead to biased results. This explains the degradation of the RE and CC metrics in terms of electrical potential for the experimental data compared to the simulated model.

## Author contributions

AK is the main author of the paper. She participated in the implementation of the methods. She participated in the analysis of the results. PM participated in the implementation of the different methods. She participated in the analysis of the results. LB performed the *ex-vivo* experiment. She participated in the writing of the paper. She participated in the analysis of the results. NZ is the supervisor of AK and PM. He performed the *in silico* simulations, participated in the design of the software implementing all of the methods used here. He participated in the analysis of the results. He participated in the writing of the paper.

### Conflict of interest statement

The authors declare that the research was conducted in the absence of any commercial or financial relationships that could be construed as a potential conflict of interest.
